# Cytokine Storm in COVID-19 Patients, Its Impact on Organs and Potential Treatment by QTY Code-Designed Detergent-Free Chemokine Receptors

**DOI:** 10.1155/2020/8198963

**Published:** 2020-09-23

**Authors:** Mujahed I. Mustafa, Abdelrahman H. Abdelmoneim, Eiman M. Mahmoud, Abdelrafie M. Makhawi

**Affiliations:** ^1^Department of Biotechnology, University of Bahri, Khartoum, Sudan; ^2^Faculty of Medicine, Alneelain University, Khartoum, Sudan; ^3^Department of Immunology, Ahfad University for Women, Khartoum, Sudan

## Abstract

The novel coronavirus is not only causing respiratory problems, but it may also damage the heart, kidneys, liver, and other organs; in Wuhan, 14 to 30% of COVID-19 patients have lost their kidney function and now require either dialysis or kidney transplants. The novel coronavirus gains entry into humans by targeting the ACE2 receptor that found on lung cells, which destroy human lungs through cytokine storms, and this leads to hyperinflammation, forcing the immune cells to destroy healthy cells. This is why some COVID-19 patients need intensive care. The inflammatory chemicals released during COVID-19 infection cause the liver to produce proteins that defend the body from infections. However, these proteins can cause blood clotting, which can clog blood vessels in the heart and other organs; as a result, the organs are deprived of oxygen and nutrients which could ultimately lead to multiorgan failure and consequent progression to acute lung injury, acute respiratory distress syndrome, and often death. However, there are novel protein modification tools called the QTY code, which are similar in their structure to antibodies, which could provide a solution to excess cytokines. These synthetic proteins can be injected into the body to bind the excess cytokines created by the cytokine storm; this will eventually remove the excessive cytokines and inhibit the severe symptoms caused by the COVID-19 infection. In this review, we will focus on cytokine storm in COVID-19 patients, their impact on the body organs, and the potential treatment by QTY code-designed detergent-free chemokine receptors.

## 1. Introduction

In the seafood market in Wuhan, China, a novel virus has emerged in December 2019, which was later named severe acute respiratory syndrome coronavirus 2 (SARS-CoV-2). There is still a debate about the source of this virus; nevertheless, bats are the most likely source since it is a well-known natural reservoir of a diversity of corona viruses [1–4]. The initial name of the disease was 2019-nCoV acute respiratory disease, then later, the World Health Organization (WHO) officially named the disease as coronavirus disease 2019 (COVID-19). After the local spread throughout China, the disease spread rapidly across the globe. Hence, on 30 January 2020, WHO officially declared that the COVID-19 has become a pandemic and it is to be considered as public health emergency of international concern [5, 6].

The approximate mean incubation period of SARS-CoV-2 is 5.1 days [7]. Interestingly, not all COVID-19 patients develop the same symptoms, but the immunological determinants of a poor prognosis are unknown. More than 50% of patients with SARS-CoV-2 showed no signs of fever before administrated a prober healthcare [8]. Strikingly, COVID-19 can be transmitted by asymptomatic patients, who show no inflammatory, respiratory or other organs symptoms and who also have normal chest computed tomography (CT) [9, 10] which hinder the effort to prevent the spread of COVID-19. The situation is further complicated by the observation that SARS-CoV-2 can be transmitted through the aerosols, and it can remain infectious up to 7 days on the surfaces [11]. Despite the fact that the fever and respiratory symptoms are the most common type of presentations, yet a recent report from Shandong, China, disclosed that a subset of patients did not suffer from these kind of symptoms, but rather had predominately neurologic symptoms [12–14]; more young people infected with COVID-19 are dying of strokes in their 30s–40s, while the average age of people who have strokes is usually 74. Moreover, they did not show signs of severe infections or in some cases no sign at all. SARS-CoV-2 causes large vessel occlusions (LVOs) in some of COVID-19 patients; this can ultimately lead to death [15, 16]. How exactly the virus causes blood clots is still unclear. Some researchers believe it could be because of cytokine storm while others believe that SARS-CoV-2 disrupts the function of angiotensin converting enzyme 2 (ACE2) which causes imbalance in the renin-angiotensin-aldosterone system [17]. Interestingly, and although there are many vaccine candidates under study [18–24], no effective treatment or vaccine has been developed so far [25–27].

Lu et al. provide direct evidence that the SARS-CoV-2 has recently developed mutations capable of significantly altering its pathogenicity. However, novel coronavirus still gains entry into humans by targeting ACE2 receptor that is found on lung cells, which destroy human lungs through cytokine storms, and this leads to hyperinflammation, forcing the immune cells to destroy healthy cells, which could be the reason behind COVID-19 patients' frequent intensive care admission [28]. This review deals with cytokine storm in COVID-19 patients, their impact on the organs, and the potential treatment by QTY code-designed detergent-free chemokine receptors.

## 2. Cytokine Storm and Multiorgan Failure

Cytokine storm is considered to be one of the major causes of multiple-organ failure in COVID-19 infections. Excessive infiltration of the inflammatory cells like monocyte and neutrophil into lung tissue will lead to lung injury. Another source of damage to the lung is through cytokine-induced apoptosis of lung epithelial cells.

IFN-*αβ* and IFN-*γ* induce inflammatory cell infiltration through two major mechanisms involving Fas–Fas ligand (FasL) or TRAIL–death receptor 5 (DR5) and cause the apoptosis of airway and alveolar epithelial cells. This will lead to alveolar edema and hypoxia and hence cause acute respiratory distress syndrome (ARDS) [29]. In cytokine storm, the following cytokine levels are elevated IL-1b, IL-2, IL-7, IL-8, IL-9, IL-10, IL-17, G-CSF, GMCSF, IFNg, TNFa, IP10, MCP1, MIP1A, and MIP1B [30, 31], which are associated with the increased severity of the disease [32], along with the development of ARDS and cardiac injury in patients with underling heart problems [33].

### 2.1. Cardiac Damage

Patients with underling cardiovascular condition are vulnerable to cardiac injury and irregular heart rhythm, even more, some patients who have no recorded history of heart disease still develop cardiac injury when became infected with SARS-CoV-2 due to the damage by the induced cytokines [34]. Cytokines are also implicated in developing myocarditis and pericarditis in COVID-19 patients, which is probably why treatment with cytokine inhibitor (like IL-6-targeting therapies) in these patients has shown promising results [35, 36].

In a retrospective cohort study was done by Zhou et al. in Wuhan, China, involving 191 COVID-19 patients. Increased high-sensitivity cardiac troponin I during hospital admission was noticed in more than half of those who died [37]. Furthermore, it was found that d-dimer rise above 1 *μ*g/mL is associated with fatal outcome. Systemic proinflammatory cytokine responses are thought to be mediators of atherosclerosis leading to plaque rupture through local inflammation, induction of procoagulant factors, and haemodynamic changes, which eventually lead to ischaemia and thrombosis. In addition, the ACE2 receptor for SARS-CoV-2, which is expressed on myocytes and vascular endothelial cells, could be playing a role in myocardial injury [38–40].

### 2.2. Acute Respiratory Distress Syndrome (ARDS)

Pneumonia associated with COVID-19 could be complicated by acute respiratory disease syndrome which is confirmed by the appearance of bilateral glass appearance on the computer tomography [41].

The pathophysiology of COVID-19-associated ARDS has a similarity to that of severe community-acquired pneumonia induced by other viruses [42]. Activation of coagulation pathways in cytokine storm syndrome will lead to progressive lung injury. Furthermore, thrombin plays a vital role in promoting clot formation and preventing bleeding, but another significant role is the augmentation of inflammation via proteinase-activated receptors (PARs), particularly PAR-1, which is why PAR-1 antagonists is a promising mode of treatment in alleviating the lung damage associated with cytokine storm. Thrombin generation is regulated by many factors, such as antithrombin III, tissue factor pathway inhibitor, and the protein C system, which all became impaired during inflammation leading to the formation of microthrombosis and acute lung injury [43, 44].

### 2.3. Renal

The incidence of acute kidney injury in COVID-19 patients was estimated to be up to 5%. It is more common in the intensive care setting and could be considered as a poor prognostic factor for survival [45].

The inflammatory response associated with cytokine storm will lead to hypoperfusion injury to the renal tubules coupled with increased vascular permeability and cardiomyopathy, which may lead to developing cardio renal syndrome type 1, a condition characterized by pleural effusions, edema, intravascular fluid depletion, and hypotension [46]. In addition, direct cytopathic damage caused by SARS-CoV-2 is thought to be one of the underlying mechanisms of renal damage associated with COVID-19 [47, 48].

### 2.4. Liver

It was thought that COVID-19 causes direct liver injury through viral hepatitis, which is accompanied by the rise in bilirubin and aminotransferase levels in COVID-19 patients [49, 50], yet studies suggested that clinical liver injury is uncommon on the course of the disease. So it is possible that elevated liver enzymes may not be from the liver alone, and confounding factors like myositis could be the cause behind this rise [51].

### 2.5. Central Nervous System

In a retrospective, observational case series, involving 214 patients with COVID-19, neurological manifestations like headache, ataxia, and seizure were noticed in 36.4% of them. These manifestations were more common in patients with severe forms of the disease. These symptoms could be due to ACE receptor involvement, in addition to the elevated proinflammatory cytokines in serum associated with cytokine storm, which may lead to neurological symptoms due to skeletal muscle damage [52, 53].

## 3. Immune Dysfunction

Peripheral CD4 and CD8 T cells showed hyperactivation in severe COVID-19 patients. This high concentration of proinflammatory CD4 T cells and cytotoxic granule CD8 T cells is suggestive of antiviral immune responses and overactivation of T cells [54]. Furthermore, numerous studies have described lymphopenia as a common feature of COVID-19 [34, 55]. All these dynamic factors may account for the severity and mortality associated sometimes with the disease.

## 4. The QTY-Modified Protein Concept

Zhang et al. designed a novel protein called QTY code, through which hydrophobic amino acids leucine (L), isoleucine (I), valine (V), and phenylalanine (F) are replaced by glutamine (Q), threonine (T), and tyrosine (Y). “The QTY code is based on the fact that the electron density map of hydrophobic L is similar to that of hydrophilic N and Q; the electron density maps of hydrophobic I and V are similar to that of hydrophilic T; and the electron density map of hydrophobic F is similar to that of the hydrophilic Y” [56]. QTY variant receptors have almost the same physiological characteristics to those of innate receptors without the existence of hydrophobic sides [57]. Currently, there are 20 Fc-fusion proteins in demand [58], although there have been reported several dissolvable proteins in the innate form [58, 59].

Levin et al. bioengineered the QTY variants to considerably increase the half-life of the designed protein in human plasma. It can also enhance the welfare of the fused proteins as a result of decreased immunogenicity [60].

**Figure 1:**
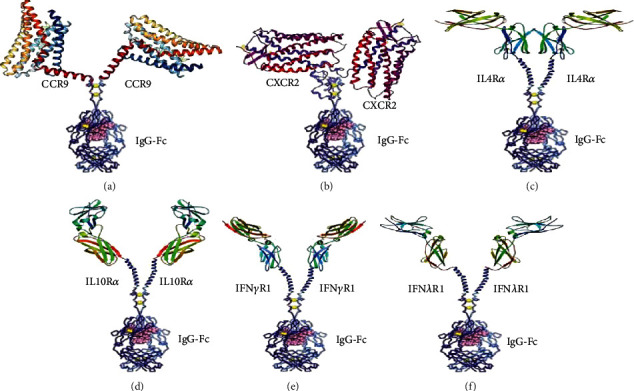
Shows a design for Fc-fused QTY receptors: (a) CCR9QTY-Fc; (b) CXCR2QTY-Fc; (c) IL4R*α*QTY-Fc; (d) IL10R*α*QTY-Fc; (e) IFN*γ*R1QTY-Fc; (f) IFN*λ*R1QTY-Fc.

Zhang et al. “described the application of the QTY code on six variants of cytokine receptors, including interleukin receptors IL4*α*R and IL10*α*R, chemokine receptors CCR9 and CXCR2, as well as interferon receptors IFN*γ*R1 and IFN*λ*R1” (Figure 1). The QTY receptors may be engineered to facilitate to cure autoimmune diseases, infectious diseases, and cancers. The QTY receptors are engineered simply to make easy to redesigned and used widely [56].

## 5. Treatment of Cytokine Storm by QTY Code-Designed Detergent-Free Chemokine Receptors

Cytokine storm is the leading side effect of cellular immunotherapy, which is potentially a life-threatening event. It can also be triggered by viral infections such as COVID-19 infections. COVID-19 triggers cytokine storm in many stages of its pathological course that causes lung fibrosis, acute respiratory distress syndrome, and eventually leads to multiorgan failure [34, 54, 61]. That is why to alleviate the symptoms and treat the disease, it is vital to remove excessive cytokines efficiently and rapidly [33].

At early stages of COVID-19 pandemic, Hao et al. tested their Fc-fusion water-soluble receptors to see if it has any influence to COVID-19; they found that it inhibits the excessive cytokines released during CAR-T treatment, therefore reducing organ damage and toxicity. Cytokine receptor–Fc-fusion proteins serve as an antibody-like decoy to dampen the excessive cytokine levels associated with cytokine storm in COVID-19 infection [57].

## 6. Alternative Treatment Approaches with Cytokine Storm

### 6.1. Stem Cell Therapy

In addition to self-renewal and differentiation, mesenchymal stem cells (MSCs) have anti-inflammatory and immune regulatory roles, as it can inhibit the secretion of proinflammatory cytokines, such as IL-1, TNF-*α*, IL-6, IL-12, and IFN-*γ*, and hence suppressing the activation of cytokine storms [62, 63]. Furthermore, MSC can secrete IL-10, keratinocyte, hepatocyte, and vascular endothelial growth factors, which collectively will help in resisting the formation of fibrosis and in the repairing of damaged lung tissues [64]. MSC seems to be an effective strategy for the treatment cytokine storm.

### 6.2. The Artificial Liver Technology

Another interesting treatment modality is the blood purification treatments such as plasma exchange and filtration that are currently used in some hospitals, which can reduce inflammation, through the removal of inflammatory elements and thus preventing the formation of cytokine storm in COVID-19 patients [65, 66].

### 6.3. Chloroquine

Chloroquine is thought to suppress the cytokine storm in COVID-19 patients through inhibiting the production of inflammatory mediators like TNF and IL-6. Building on the early positive reports, chloroquine phosphate has been used successfully in the treatment of adult COVID-19 patients in China with varied success rate [67]. The recommended dose in one of the associated clinical trials was as follows: “If the weight is more than 50 kg, 500 mg each time, 2 times a day, 7 days as a treatment course; If the weight is less than 50 kg, 500 mg each time on the first and second days, twice a day, 500 mg each time on the third to seventh days, once a day” [67].

### 6.4. IFN-*λ*

IFN-*λ* mainly acts on epithelial cells and diminishes the mononuclear macrophage-mediated proinflammatory activity of IFN-*αβ* [68]. Furthermore, IFN-*λ* inhibits the recruitment of the early inflammatory cells (neutrophil) to the inflammation sites [69]. It is known that SARS-CoV-2 primarily infects alveolar epithelial cells (AEC), and that IFN-*λ* can trigger the antiviral genes in these cells, thus improving the antiviral effects against the virus. Although interferons can reduce the viral load and improves the symptoms of patients [70–72], still, it fails to reduce mortality rates [72].

## 7. Advantages and Limitations of Cytokine Storm Inhibition by QTY Code-Designed Detergent-Free Chemokine Receptors

The main advantage of QTY receptors is to serve as an antibody-like structure to decrease the excessive cytokine levels related with cytokine storm syndrome in COVID-19 infection.

Levin et al. bioengineered the QTY variants to considerably increase the half-life of the designed protein in human plasma. It can also enhance the welfare of the fused proteins as a result of decreased immunogenicity [60].

Another advantage is that the Fc region can be engineered and used widely. QTY receptors, particularly CCR9 and CXCR2, are distinctive due to it provides a novel relatively simple stage for additional design of QTY receptors for therapeutic purposes [57].

The main limitation of QTY code-designed detergent-free chemokine receptors is that it had been tested only in mice, while the human clinical trials had just been recently started in April 2020 [57].

## 8. Conclusion

Cytokine storm is the leading side effect during cellular immunotherapy which is a potentially life-threatening occurrence. It can also be triggered by viral infections such as COVID-19 infections. Importantly, COVID-19 has many consequences induced by the cytokine storm in many stages of its pathological course which include lung fibrosis, acute respiratory distress syndrome, and multiorgan failure leading eventually to the death of the patient. To alleviate the symptoms, it is vital to inhibit cytokine storm efficiently and rapidly. The QTY-modified proteins could provide a solution to these excess cytokines. These synthetic proteins can be injected into the body to bind the excessive cytokines generated by the cytokine storm; this will eventually remove them from the circulation and probably inhibit the severe complications caused by the COVID-19 infection.
